# Central acetabular osteophytes (CAO) are more prevalent in the borderline developmental dysplastic hip (BDDH) patients: a propensity-score matched CT study

**DOI:** 10.1186/s13018-022-03056-x

**Published:** 2022-03-12

**Authors:** Fan Yang, Hong-Jie Huang, Zi-Yi He, Yan Xu, Xin Zhang, Jian-Quan Wang

**Affiliations:** Department of Sports Medicine, Peking University Third Hospital. Institute of Sports Medicine of Peking University. Beijing Key Laboratory of Sports Injuries, 49 North Garden Rd, Haidian District, Beijing, 100191 People’s Republic of China

**Keywords:** Borderline developmental dysplastic hip, Central acetabular osteophytes, Central acetabular decompression, Hip CT

## Abstract

**Background:**

The acetabular fossa often showing the first signs of degeneration, Central acetabular osteophytes (CAO) have been increasingly recognized during hip arthroscopy. The purpose of this study was to investigate the condition of CAO in BDDH hips and compare cotyloid fossa size between the BDDH and the non-BDDH hips on CT images.

**Methods:**

We performed a retrospective analysis of prospectively collected data of hip CT images of FAI or labral injury patients. A 1:2 propensity-score matched observational study comparing the linear length of cotyloid fossa was analyzed. Cotyloid fossa width (CFW) and cotyloid notch width (CNW) were measured on axial images, cotyloid fossa height (CFH) and cotyloid fossa depth (CFD) were measured on coronal images. Within the CAO patients, we performed central acetabular decompression (CAD) and then observed the morphology change in fossa.

**Results:**

Propensity-score matching yielded 61 BDDH hips and 122 non-BDDH hips. BDDH hips had a higher prevalence of CAO and a decreased linear length of cotyloid fossa (CFW, CFH and CNW). In the BDDH group, 33 hips underwent CAD, postoperative CFW, CFH and CNW were significantly increased (*p* < .001 for all), and had no statistical difference compared with the non-BDDH hips (*p* = .193, *p* = .132, *p* = .421, respectively).

**Conclusion:**

BDDH hips had a significantly higher prevalence of CAO than adequate acetabular coverage hips. After the procedure of CAD, BDDH hips were found to have acetabular parameters (CFW, CFH, CNW) and were restored to that of the control hips.

## Background

Developmental dysplasia of the hip (DDH) can lead to multiple conditions, such as labral lesions, chondral and ligamentum teres damage, which may contribute to the development of early osteoarthritis [1]. The severity of acetabular dysplasia can be classified into mild, moderate, and severe based on the lateral center edge angle (LCEA). Borderline developmental dysplastic hip (BDDH), with mild acetabular undercoverage, was first described by Fredensborg [2]. BDDH frequently coexists with intra-articular chondrolabral pathology and cam-type femora-acetabular impingement (FAI) [3]. The direct cause of symptoms in BDDH is soft tissue pathology rather than osseous structures abnormality [4]. With the recent advances in hip arthroscopy instrumentation and techniques, the surgical indications have gradually expanded, arthroscopic treatment of borderline dysplasia could provide satisfying benefits [5, 6], but the outcomes could be influenced by some risk factors [7–9], such as broken Shenton line, osteoarthritis, and Tӧnnis angle > 15°.

The contact stress distribution is an important role during the development of the human hip. The acetabulum has a cartilage void zone in its central and inferior part, creating the cotyloid fossa; this characteristic gives its articular surface a horseshoe shape and can optimize the contact stress distribution in the hip joint [10]. Despite this key role, the acetabular fossa receives relatively little attention in this discussion of hip pathology. The fossa is filled with the ligamentum teres, synovial membrane, and intra-articular adipose tissue(IAAT), which has adipocytes, fibroblasts, leucocytes, and abundant mast cells [11]. The acetabular fossa also contributes to the lubrication of the hip joint; the fossa is the only intra-articular tissue associated with fluid production, which is essential for cartilage nutrition and load transmission [12].

Lesions of the acetabular fossa are an uncommon cause of hip pain, of which the prevalence of central acetabular osteophytes(CAO) are as frequent as that of femoral head osteophytes in the degenerative changes [13]. CAO are thought to arise from the attachment of transverse acetabular ligament, progressing to involve the entire fossa [14]. CAO can create improper contact between the acetabular cartilage and femoral head by altering the congruency. CAO are significantly associated with the degree of chondral damage, which has been suggested as an early manifestation of osteoarthritis(OA) [15]. Sudsriluk et al. [16] found advanced stage of acetabular fossa change was statistically correlated with the advanced stage of acetabular cartilage degeneration. But CAO can also be seen in patients without advanced degeneration of the hip joint, central acetabular Impingement (CAI) can cause the formation of CAO in femoral-acetabular impingement (FAI) patients [17]. Cotyloid fossa lesions in the central compartment are increasingly being recognized and addressed at the time of routine hip arthroscopy [18, 19]. Arthroscopic acetabular notchplasty is a favorable method of decompressing the central acetabular region [15]. CAO patients treated with central acetabular decompression (CAD) had favorable outcomes at a minimum of 2 years follow-up [18].

At our center, we previously presenting the BDDH patients with labral tear observing that the incidence of cotyloid fossa hyperplasia and osteophyte formation is high. This precipitated an interest in whether hyperplasia of the cotyloid fossa or CAO, might have an association with BDDH. There is a paucity of literature on hyperplasia of cotyloid fossa and even less information on its relation with BDDH. The purpose of this study was to investigate the condition of CAO in the BDDH patients and compare cotyloid fossa size between the BDDH and the non-BDDH patients on CT images.

## Methods

### Patient selection

**Fig. 1:**
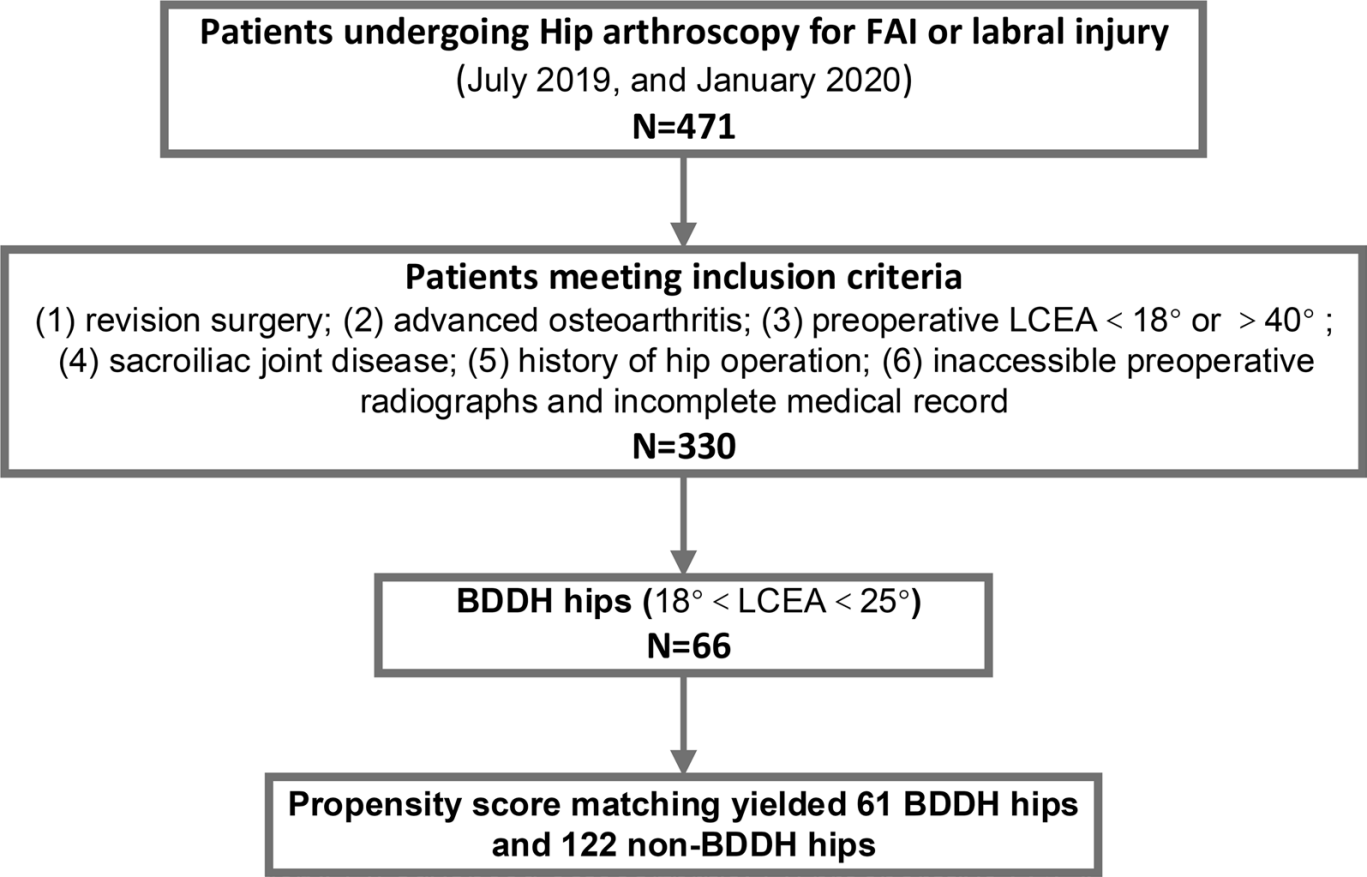
CONSORT (Consolidated Standards of Reporting Trials) diagram indicating total patient population meeting inclusion and exclusion criteria. FAI: femoral acetabular impingement. BDDH: borderline developmental dysplastic hip

With the consent of the Ethics Committee at our institution, we performed a retrospective analysis of prospectively collected data between July 2019 and January 2020. FAI or labral injury patients, age 16–50 years, were included in this study. Exclusion criteria included: (1) revision surgery; (2) advanced osteoarthritis (Tӧnnis grade ≥ 2); (3) preoperative LCEA < 20° or > 40°; (4) sacroiliac joint disease; (5) history of hip operation; (6) inaccessible preoperative radiographs and incomplete medical record. Patients underwent preoperative anteroposterior (AP) pelvis and Dunn lateral radiography. The LCEA angle, Tӧnnis angle, and Sharp angle were measured on AP pelvis radiographs. The alpha angle was measured on Dunn lateral radiographs. The patients were classified into two groups based on the preoperative LCEA. The BDDH group had an LCEA between 20° and 25°, and the non-BDDH group had an LCEA between 25°and 40°(Fig. 1). The condition of cotyloid fossa hyperplasia and osteophyte formation was made intraoperatively and verified by surgical records and video records. In the BDDH group, patients were further classified into two groups based on the treatment of decompression of CAO.

### Surgical technique

**Fig. 2:**
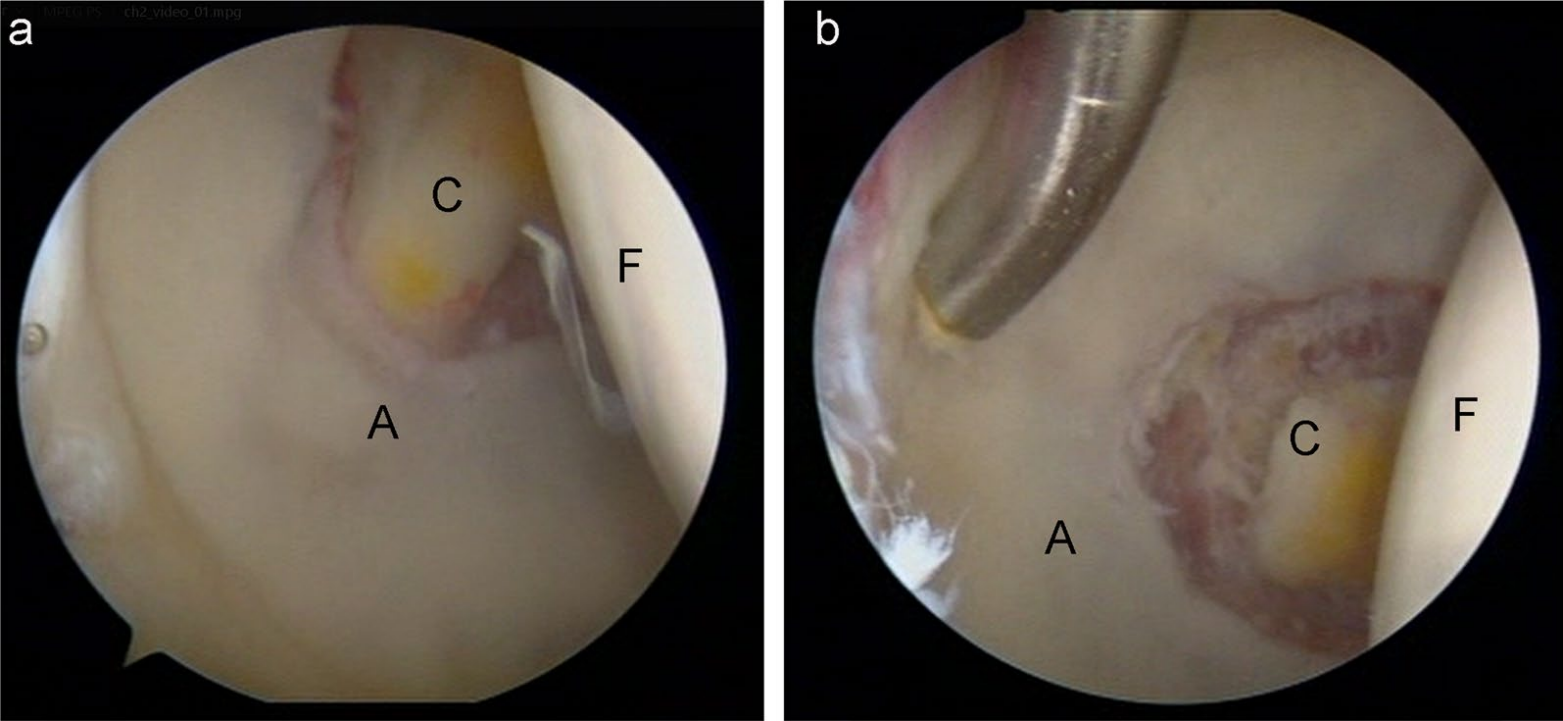
Intra-articular arthroscopic view of a central acetabular decompression (CAD). **A** Preoperative. **B** Postoperative. (A, acetabular cartilage; C, cotyloid fossa; F, femoral head)

All hip arthroscopies were performed by one senior author. The patient was placed in the modified supine position on standard hip traction. After the establishment of anterolateral (AL) portal and the midanterior portal (MAP), routine acetabuloplasty and labral repairing were performed. Then hips with CAO requiring CAD were performed in the central compartment. After identification of the morphology of cotyloid fossa, radiofrequency was used to expose the edge of hyperplastic osteophyte without disturbing ligamentum teres, and a long 4.5 mm 133 round bur (Smith & Nephew, Andover, MA) was introduced to remove the sclerotic CAI osteophyte. Capsular plication was performed for all hips after a routine femoroplasty. Figure 2 shows an arthroscopic view of the cotyloid fossa of a left hip with CAO. The intraoperative data were documented including the procedures of the ligamentum teres, labral repair, femoroplasty, and acetabuloplasty.

### CT measurement protocol

Compared to MRI, CT scan is a more accurate imaging modality for bone assessment. At our center, a preoperative CT scan was routinely performed. The GE Light speed 64 slice spiral CT (GE Medical System, Chalfont St Giles, UK) was used for CT examination. The collimator width was 0.625, the pitch was 1.0; the slice thickness of reconstruction was 3 mm, and the interlayer distance was 3 mm. Axial, coronary, and sagittal scanning were routinely performed.

**Fig. 3:**
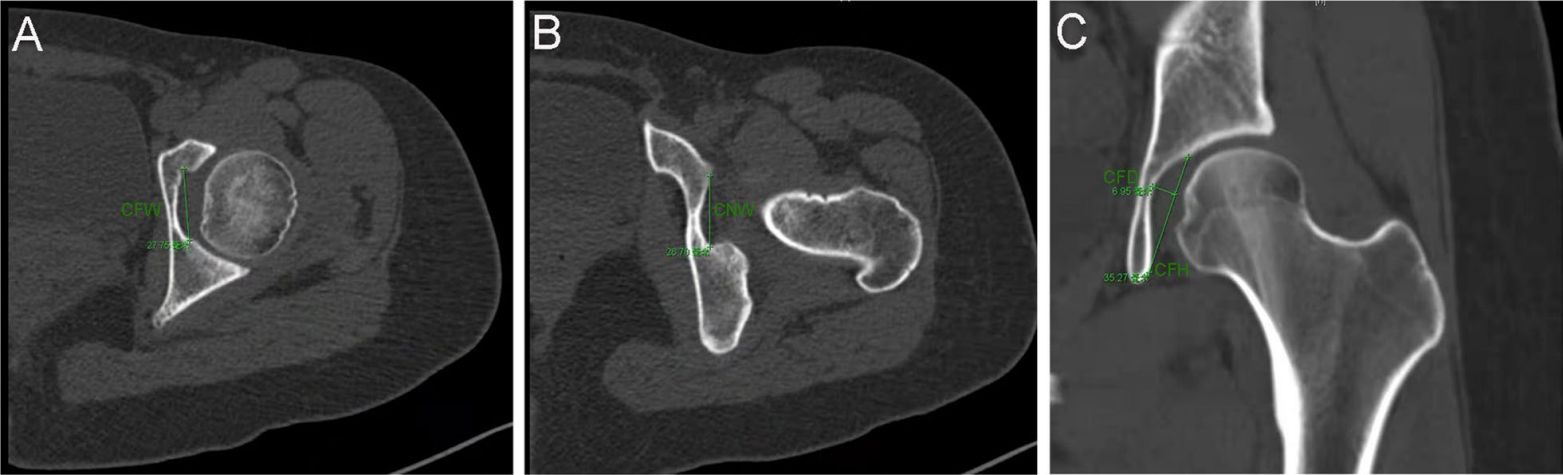
The measurement of the linear length of cotyloid fossa on CT images of the left hip. **a** and **b** CFW and CNW were measured on axial images; **c** CFH and CFD were measured on coronal images. CFW = Cotyloid Fossa Width; CFH = Cotyloid Fossa Height; CFD = Cotyloid Fossa Depth; CNW = cotyloid notch Width

Radiographic linear length measurements were performed using a picture archiving and communication system (PACS; GE Healthcare). The Cotyloid Fossa Width (CFW), Cotyloid Fossa Height (CFH), Cotyloid Fossa Depth (CFD), cotyloid notch Width (CNW) were measured on three or more adjacent CT scan images and the biggest value was selected for these paraments. CFW and CNW were measured on axial images. CFW was measured from the anterior extent to the posterior extent of the fossa. CNW was measured from the anterior extent to the posterior extent of the acetabular notch. CFH and CFD were measured on coronal images. CFH was measured from the top of the cotyloid fossa to the most inferior portion of the bony fossa. CFD was the longest perpendicular distance from the acetabular opening plane to the medial acetabular wall (Fig. 3). In the BDDH group, we further measured CFW, CFH, and CNW on the patient’s postoperative CT. Two orthopedic surgeons measured all parameters under the supervision of a senior radiologist. Both observers were blinded to all clinical data of patients. They performed two measurements one month apart to determine the reliability and obtain clinically meaningful results.

### Statistical analysis

A 1:2 propensity-score match based on age, gender, BMI, unilateral or bilateral symptoms was performed using RStudio to control for potential confounding variables in the BDDH group and non-BDDH group. Data for CFW, CFH, CFD, CNW were confirmed to be normal by the Kolmogorov–Smirnov test. The student’s unpaired t-test was performed to compare the differences in CFW, CFH, CFD, CNW between the 2 groups. C-squared test was used to compare categorical data in intraoperative procedures. Paired t-test was used to compare CFW, CFH, and CNW preoperative to postoperative in patients who underwent CAD in the BDDH group. All statistical analyses were performed using SPSS version 26 (IBM, Armonk, NY) with a statistical significance set at *p* < 0.05.

## Results

A total of 471 patients underwent a hip arthroscopy during the study period. We identified 330 patients who met both our inclusion and exclusion criteria. among them, 66 hips with BDDH. Propensity score matching yielded 61 BDDH hips and 122 non-BDDH hips, their mean age was 36.5 years (range 16–50 years). After matching, there were no significant differences in age, sex, BMI, and laterality between the two groups. The preoperative radiographic findings for both groups were described in Table 1. The mean LCEA angle was 23.0° in the BDDH group and 33.2° in the non-BDDH group. The BDDH group also had a larger Tӧnnis angle and Sharp angle, indicating the deficient coverage of acetabular [20].

**Table 1:** Patient characteristics

Category	BDDH	Non-BDDH	*p* Value
No. of hips	61	122	
Age, yr	35.8 ± 9.0	36.9 ± 8.7	.412
BMI, kg/m2	22.8 ± 3.1	23.00 ± 3.0	.710
Sex, n (%)			> .99
Female	35(57.4)	70(57.4)	
Male	26(42.6)	52(42.6)	
Laterality, n (%)			.748
Left	23(37.7)	49(40.2)	
Right	38(62.3)	73(59.8)	
Uni-or bilateral,n (%)			.857
Unilateral	55(90.2)	111 (91.0)	
Bilateral	6 (9.8)	11(9.0)	
LCEA angle	23.0 ± 1.8	33.2 ± 4.5	< .001*
Alpha angle	63.0 ± 8.5	59.1 ± 8.7	.05
Tӧnnis angle	12.2 ± 4.3	4.6 ± 5.1	< .001*
Sharp angle	43.4 ± 2.5	38.8 ± 2.7	< .001*

*p value < .05

Values are given as mean ± SD. BDDH, Borderline developmental dysplastic hip; BMI, body mass index

**Fig. 4:**
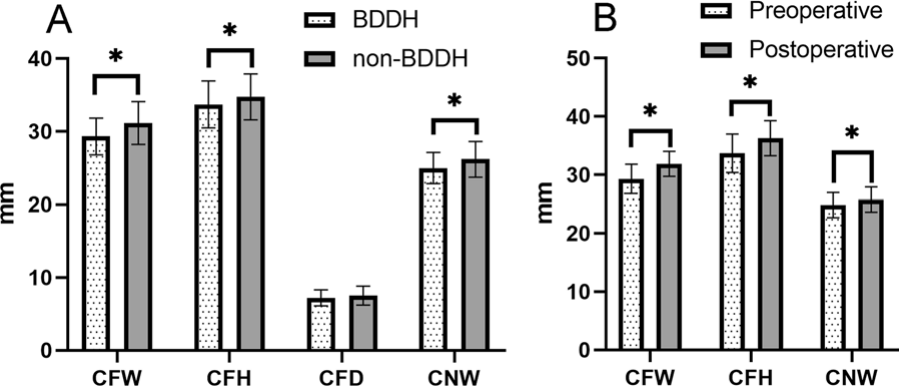
The comparison of the linear length of Cotyloid Fossa. **a** The white bar in the figure indicates the BDDH group, and the gray bar indicates the non-BDDH group; **b**. The white bar in the figure indicates preoperative in the BDDH group, and the white bar indicates postoperative in the BDDH group. CFW = Cotyloid Fossa Width; CFH = Cotyloid Fossa Height; CFD = Cotyloid Fossa Depth; CNW = cotyloid notch Width. CAD = central acetabular decompression. BDDH: borderline developmental dysplastic hip

The BDDH group had a decreased absolute CFW size compared to the control group (29.31 ± 2.51 mm versus 31.15 ± 2.94 mm, *p* < 0.001). The BDDH group had lower CFH measurements compared with the control group (33.69 ± 3.21 mm versus 34.73 ± 3.14 mm, *p* = 0.037). There was a significant difference in the height-width ratio between the two groups (1.15 ± 0.11 versus 1.12 ± 0.10, *p* = 0.044). The BDDH group also had lower CNW measurements compared with the control group (24.96 ± 2.16 mm versus 26.17 ± 2.45 mm, *p* = 0.01) (Fig. 4). There was no difference in CFD measurements between the two groups (Table 2).

**Table 2:** Summary measurements by group

	BDDH	Non-BDDH	*p* Value
CFW	29.31 ± 2.51	31.15 ± 2.94	< .001*
CFH	33.69 ± 3.21	34.73 ± 3.14	.037*
CFH: CFW	1.15 ± 0.11	1.12 ± 0.10	.044*
CFD	7.21 ± 1.09	7.51 ± 1.29	.117
CNW	24.96 ± 2.16	26.17 ± 2.45	.01*

Data are presented as mean ± SD in millimeters. BDDH, Borderline developmental dysplastic hip; CFW = Cotyloid Fossa Width; CFH = Cotyloid Fossa Height; CFD = Cotyloid Fossa Depth; CNW = cotyloid notch Width. *p value < .05

Overall, most of the study population underwent labral repair, femoroplasty, and acetabuloplasty. The BDDH group had a significantly higher number of CAO (54.1% vs 30.3% *p* = 0.02) and ligamentum teres treatment (*p* = 0.01). There were no differences in other intraoperative procedures between the groups.

In the BDDH group, 33 hips with underwent CAD, postoperative CFW (31.87 ± 2.14 mm versus 29.29 ± 2.18 mm), CFH (36.27 ± 3.00 mm versus 33.68 ± 3.29 mm) and CNW (25.79 ± 2.15 mm versus 24.84 ± 2.14 mm, *p* < 0.001 for all) were significantly increased. Patients who underwent CAD also had increased postoperative CFW and CFH compared with non-CAD patients in the BDDH group (*p* = 0.01, *p* = 0.02, respectively) (Table 3). Moreover, after the procedure of CAD, the BDDH patients’ postoperative size of CFW, CFH, and CNW had no statistical difference compared with the non-BDDH group (*p* = 0.193, *p* = 0.132, *p* = 0.421, respectively). The intraobserver and interobserver reliability, evaluated by the ICC, was more than 0.85 (0.873–0.924) for each parameter, indicating an acceptable level of reliability.

**Table 3:** Preoperative to postoperative changes in the BDDH group

	CAD	Non-CAD	*p* Value
CFW			
Preoperative	29.29 ± 2.18	29.33 ± 2.90	.957
Postoperative	31.87 ± 2.14	29.54 ± 2.89	.01*
*p* value (pre-post)	< .001*	.114	
CFH			
Preoperative	33.68 ± 3.29	33.71 ± 3.18	.972
Postoperative	36.27 ± 3.00	33.65 ± 3.27	.02*
*p* value (pre-post)	< .001*	.406	
CNW			
Preoperative	24.84 ± 2.14	25.09 ± 2.22	.654
Postoperative	25.79 ± 2.15	25.07 ± 2.21	.201
*p* value (pre-post)	< .001*	.776	

Data are presented as mean ± SD in millimeters; CFW = Cotyloid Fossa Width; CFH = Cotyloid Fossa Height; CFD = Cotyloid Fossa Depth; CNW = cotyloid notch Width. CAD = central acetabular decompression. *p value < .05

## Discussion

The results showed that the BDDH patients had a high prevalence of CAO. The BDDH group had a decreased linear length of Cotyloid Fossa Width (CFW), Cotyloid Fossa Height (CFH), and cotyloid notch Width (CNW) size compared to the non-BDDH group. In the BDDH group, CFW, CFH, and CNW were significantly increased after the decompression of CAO, the increase after CAD brought the measurements in line with the non BDDH group.

The acetabular fossa is a distinctive area in which osteophytes develops, CT is recommended for assessing the morphology of the acetabular fossa. On an anteroposterior pelvic radiograph, the acetabular fossa extends from the teardrop to an ill-defined area between the medial sourcil and the superior edge of the fovea capitis [11, 20], CAO in advanced osteoarthritis patients can be detected as saber-tooth bony excrescence on cross-sectional imaging, or cause the buttressing effect medial to the head of the femur and yield a double teardrop view in radiographs [21]. But the mild CAO may easily be neglected in the plain radiograph, they are more likely to lie inside the joint, not visible on conventional radiographs.

Previous studies focused mainly on the size of acetabulm and the result of the acetabular fossa size varied considerably. A three-dimensional (3D) CT study described that the dysplastic acetabula were elongated in females but the width was similar in both females and males [22]. Steppacher et al. [23] found dysplastic hips have a decreased size of the lunate surface, a decreased outer acetabular rim, but an increased acetabular fossa. Stephanie et al. [24] measured linear length dimensions of the cotyloid fossa in normal and dysplastic hips on MRI and CT, they found dysplastic acetabula had smaller CFH and CFW compared with normal acetabula. But the sample size was relatively small (20 hips) and they did not report if there were CAO formation in both groups. CAO progressing to involve cotyloid fossa circumferentially and may contribute to the smaller size of cotyloid fossa in the BDDH patients. Our result showed that the BDDH patients had a smaller size of CFW, CFH, and CNW compared with the control group. Moreover, after CAD, there is no statistical difference in CFW, CFH, and CNW size compared with the non-BDDH group.

Our result showed that the prevalence of CAO is high in the BDDH patients compared with the control group. Parth et al. [14] described CAI is associated with femoral head and ligamentum teres damage, they postulated CAI plays an important role in the formation of CAO. Different from FAI, the abnormal contact of CAI happens between cotyloid fossa and adjacent tissue (femoral head and the ligamentum teres), rather than the acetabular margin and the junction of the femoral head and neck. The ligamentum teres is thought to be an important hip stabilizer, especially in dysplasia hips [25]. Ippolito et al. [26] reported a thicker and longer ligamentum teres with wide areas of fibro-cartilaginous metaplasia in dysplastic hips, leading to anterosuperolateral migration of the femoral head, this migration causes distraction of the femoral head and increases the tension on ligamentum teres [14]. Our result showed that the BDDH patients had a significantly higher number of ligamentum teres treatment. Based on previous studies and our result, we conclude that the BDDH patients may have a higher incidence ligamentum teres damage.

The relationship between CAO and OA remains controversial, Varich et al. [27] used “the Saber tooth sign” to describe CAO and considered it as an early manifestation of OA. Asheesh [14] et al. described CAO are associated with degenerative ligamentum teres and femoral head damage arthroscopy. However, a systematic review concluded there is no association between the progression of hip OA and acetabular osteophytes [28]. Although our results showed that CAO is more prevalent in the BDDH patients, a CT study suggested that there is no association between BDDH and the pathogenesis of OA [29]. During the procedure of arthroscopy, we found inflammatory manifestation around IAAT tissue within the fossa. Sampatchalit et al. [16] found gradual acetabular fossa degenerative changes in cadaveric specimens with osteoarthritic changes, included a decrease volume of the adipose tissue, fibrocartilaginous metaplasia, and calcifications. Hence, we need a prognostic study focused on the comparison between patients undergoing CAD and those treated conservatively for CAO.

There are some limitations to our study. First, we did not analyze the differences in cotyloid fossa size between male and female hips. Male acetabula are larger in general; however, we controlled the effects of sex by including even numbers of male and female subjects in the two groups. Second, patient-reported outcomes (PROs) were not reported in this cross-sectional study. Although previous studies had reported the favorable outcomes of CAD at minimum 2-Year follow-up [18]. Markus et al. [17] reported that CAO are associated with an unfavorable outcome after arthroscopy in young FAI patients, and they considered hips with CAO as ‘‘hips at risk.’’ The long-term follow-up data and the long-term effect of CAD need to be further evaluated.

## Conclusion

BDDH hips had a significantly higher prevalence of CAO than adequate acetabular coverage hips. After the procedure of CAD, BDDH hips were found to have acetabular parameters (CFW, CFH, CNW) and were restored to that of the control hips.

## Data Availability

The datasets used and/or analyzed during the current study are available from the corresponding author on reasonable request.

## Abbreviations

CAO: Central acetabular osteophytes
CAD: Central acetabular decompression
CFW: Cotyloid fossa width
CNW: Cotyloid notch width
CFH: Cotyloid fossa height
CFD: Cotyloid fossa depth
DDH: Developmental dysplasia of the hip
BDDH: Borderline developmental dysplastic hip
FAI: Femoraacetabular impingement
